# Applying a One Health Approach in Global Health and Medicine: Enhancing Involvement of Medical Schools and Global Health Centers

**DOI:** 10.5334/aogh.2647

**Published:** 2021-03-26

**Authors:** Catherine Machalaba, Jill Raufman, Assaf Anyamba, Amanda M. Berrian, Franck C. J. Berthe, Gregory C. Gray, Olga Jonas, William B. Karesh, Michelle H. Larsen, Ramanan Laxminarayan, Lawrence C. Madoff, Keith Martin, Jonna A.K. Mazet, Elizabeth Mumford, Tina Parker, Lilian Pintea, Melinda K. Rostal, Rafael Ruiz de Castañeda, Neil M. Vora, Chadia Wannous, Louis M. Weiss

**Affiliations:** 1 EcoHealth Alliance, 520 Eighth Ave, Ste 1200, New York, NY, USA; 2 Future Earth oneHEALTH global research project, New York, NY, USA; 3 Albert Einstein College of Medicine, Bronx, NY, USA; 4 Goddard Earth Science and Technology Research (GESTAR) Universities Space Research Association, NASA/Goddard Space Flight Center, Greenbelt, MD, USA; 5 College of Veterinary Medicine, The Ohio State University, Columbus, Ohio, USA; 6 World Bank Group, Washington, D.C., USA; 7 Duke University School of Medicine & Global Health Institute, Durham, NC USA; Duke-NUS Medical School, Singapore; and Duke Kunshan University, Kunshan, China; 8 Harvard Global Health Institute, Cambridge, MA, USA; 9 Center for Disease Dynamics, Economics & Policy (CDDEP), Washington, D.C., USA; 10 ProMED, International Society for Infectious Diseases, Brookline, MA, USA; 11 Consortium of Universities for Global Health, Washington, D.C., USA; 12 One Health Institute, School of Veterinary Medicine, University of California, Davis, CA, USA; 13 Country Health Emergency Preparedness and IHR, World Health Organization, Geneva, Switzerland; 14 Office of Biodefense, Research Resources, and Translational Research, DMID, NIAID, NIH, Bethesda, MD, USA; 15 Jane Goodall Institute, Vienna, VA, USA; 16 Institute of Global Health, Department of Community Health and Medicine, Faculty of Medicine, University of Geneva, Switzerland; 17 NYC Dept of Health and Mental Hygiene, New York, NY, USA; 18 Career Epidemiology Field Officer Program, Centers for Disease Control and Prevention, Atlanta, GA, USA; 19 Toward a Safer World Network, Future Earth Health Knowledge Action Network, Stockholm, Sweden

## Abstract

**Background:**

Multidisciplinary and multisectoral approaches such as One Health and related concepts (e.g., Planetary Health, EcoHealth) offer opportunities for synergistic expertise to address complex health threats. The connections between humans, animals, and the environment necessitate collaboration among sectors to comprehensively understand and reduce risks and consequences on health and wellbeing. One Health approaches are increasingly emphasized for national and international plans and strategies related to zoonotic diseases, food safety, antimicrobial resistance, and climate change, but to date, the possible applications in clinical practice and benefits impacting human health are largely missing.

**Methods:**

In 2018 the “Application of the One Health Approach to Global Health Centers” conference held at the Albert Einstein College of Medicine convened experts involved in One Health policy and practice. The conference examined issues relevant to One Health approaches, sharing examples of challenges and successes to guide application to medical school curricula and clinical practice for human health. This paper presents a synthesis of conference proceedings, framed around objectives identified from presentations and audience feedback.

**Findings and Recommendations:**

The following objectives provide opportunities for One Health involvement and benefits for medical schools and global health centers by: 1) Improving One Health resource sharing in global health and medical education; 2) Creating pathways for information flow in clinical medicine and global health practice; 3) Developing innovative partnerships for improved health sector outcomes; and 4) Informing and empowering health through public outreach. These objectives can leverage existing resources to deliver value to additional settings and stakeholders through resource efficiency, more holistic and effective service delivery, and greater ability to manage determinants of poor health status. We encourage medical and global health educators, practitioners, and students to explore entry points where One Health can add value to their work from local to global scale.

## Background

In recent years, concepts such as One Health, Planetary Health, and EcoHealth have been used to explore the complex linkages among humans, animals, plants, and the environment in relation to health and wellbeing. At their core, these concepts promote collaboration across disciplines and sectors to more holistically understand and address health threats at the human-animal-environment interface. A One Health approach has increasingly been adopted in national and international plans and strategies for zoonoses, health security, food safety, and antimicrobial resistance (AMR) and in veterinary medical education. Country-level One Health coordination mechanisms are convening relevant government agencies including sectors related to human, animal, and environmental health, as well as trade, travel, and finance and other stakeholders (e.g. development partners and media) [1]. As local, regional, and global communities face current and anticipated impacts of an ever-increasing number of changes that also drive disease risks [2], the cost of inaction is high [3,4]. Emerging and some endemic infectious disease events are on the rise; and in today’s interconnected world, a disease threat anywhere is a disease threat everywhere, as demonstrated by the COVID-19 pandemic [5]. The One Health approach will be critical for solutions to prevent, prepare for, and respond to these complex threats to health.

Hippocrates first conceived of integrated health, and animal and human comparative medicine has centuries-old foundations. Even the system for taxonomic classification of animal and plant species, including that of microbes, was conceived by physician and botanist Carl Linnaeus. Veterinarian Dr. Calvin Schwabe coined the term “One Medicine” in his landmark book published in 1964, *Veterinary Medicine and Human Health*, to emphasize the similarities between human and veterinary medicine and the need for collaboration to effectively cure, prevent, and control illnesses that affect both humans and animals [6]. More recently, the term One Health was put forward to describe the inextricable linkages among the biotic and abiotic systems and health [7,8,9]. One Health is a collaborative, multisectoral, and transdisciplinary approach—working at local, regional, national, and global levels—with the goal of achieving optimal health outcomes recognizing the interconnection between people, animals, plants, and their shared environment [10]. To date, One Health efforts have been largely championed by veterinary medicine and public health communities. While this interest is reflected in veterinary school curricula and missions, adoption of One Health education in medical schools is in early stages [11]. There is limited awareness and engagement from the human clinical medical community on One Health topics, resulting in a lack of tangible benefits. For example, surveys of clinicians in the United States (U.S.) have found low rates of environmental health knowledge and training, and lack of confidence in their knowledge of diagnostics and preventative measures for zoonoses [12,13].

Although great strides have been made to develop vaccines and medicines to prevent and treat diseases and control outbreaks, in many cases reactive outbreak response measures have come at significant cost. Embracing One Health can potentially reduce morbidity and mortality from zoonotic diseases, foodborne illnesses, AMR, and other causes including non-infectious threats such as obesity, cancers, and extreme weather injuries. Proactive health protection could ultimately lower the cost of public health delivery by shifting to prevention. This potential utility is a premise of the Global Health Security Agenda, involving nearly 70 countries, intergovernmental and non-governmental organizations and private sector partners and promoting multisectoral collaboration to prevent, detect and respond to disease threats. However, the evidence base demonstrating the possible applications and impact of One Health in clinical practice and benefits for human health is largely missing [14]. Wider adoption of One Health approaches in human clinical care will require novel competencies and non-traditional pedagogical approaches backed by solid justification for these changes in workforce development.

## Methods

To explore ways to meaningfully involve and generate value for medical and other global health professionals, in December 2018 the Albert Einstein College of Medicine with EcoHealth Alliance and the Montefiore Medical Center hosted the conference “Application of the One Health Approach to Global Health Centers” as part of its biennial conference series (presentations can be accessed at *https://einsteinmed.org/centers/global-health/*). The conference brought together experts from different areas of practice (e.g., agriculture, animal and human medicine, conservation, economics, policy) working in clinical and non-clinical settings. Continuing medical education was offered for physicians. The conference covered critical One Health issues and examined application to medical school curricula and clinical practice via experiences and examples. In this paper, we share four key objectives generated from the conference. These objectives reflect priorities and next steps identified by conference speakers in follow up to presentations, participant questions, and discussion; they are not intended to be exhaustive. As One Health applications are dynamic and contextual, the recommendations are globally-relevant but adaptable to specific situations. We encourage the widest scope of academic institutions, researchers, and medical practitioners to consider relevant topics, disciplines, stakeholders, and entry points where a One Health approach can generate value to address health challenges for the populations and patients they serve.

## Findings and Recommendations

We share the following four key objectives (***Table 1***) as opportunities to enhance One Health involvement and benefits for medical schools and global health centers, some readily available and others requiring cultivation.

**Table 1:** Key objectives identified from the “Application of the One Health Approach to Global Health Centers” conference.


OBJECTIVE	ILLUSTRATIVE APPROACHES

Improving One Health resource sharing in global health and medical education.	Increase access to and dissemination of existing guidance, tools, and information resources available through One Health communities of practice. Enlist school librarians to direct faculty and students to useful One Health resources. Post One Health content on school websites, in course syllabi, or via lectures. Utilize platforms (e.g., MOOCs) that promote equitable access to information. Employ innovative pedagogical models and assessment methods to support students on interdisciplinary teams. Incorporate relevant One Health insights on ecological and epidemiological factors shaping disease risk into tools commonly used by the medical community (e.g., Medscape).

Creating pathways for information flow in clinical medicine and global health practice.	Develop communication channels for information sharing outside of ones’ immediate sphere to ensure knowledge serves its full utility for global health. Ensure channels are in place to harness front-line observations of clinicians, veterinarians, and other health practitioners to guide appropriate reporting and response. Ensure that information channels are designed, operationalized, and maintained with workflow needs and utility for potential users including clinicians.

Developing innovative partnerships for improved health sector outcomes.	Link students and faculty from different departments via mechanisms such as joint degrees, interdisciplinary events (e.g., across medical, veterinary, and public health schools), scenario-based cases, and research or practicum exchanges. Leverage resources such as information, personnel and infrastructure from animal and environmental sectors (and vice versa) to enhance risk monitoring, implementation and surge capacity. Mobilize multi-disciplinary training, research and practice initiatives to assess and serve needs for improved health status, including at community level. Identify key competencies needed for health practitioners and entry points for applied training, including interdisciplinary approaches to problem solving.

Informing and empowering health through outreach to the public.	Empower health professionals to identify and communicate urgent threats to public health and the importance of a One Health approach to inform solutions. Utilize clear messaging on action and rationale to promote uptake and consistent understanding across sectors.


### 1) Improving One Health Resource Sharing in Global Health and Medical Education

There is an extensive information base on One Health topics, reflecting involvement of diverse organizations. Many of these resources are captured on web platforms, such as those of the One Health Commission, One Health Initiative, and One Health Platform. Others have been documented in peer-reviewed articles and grey literature [15,16]. All provide entry points to information for those interested in learning more about One Health. At global level, several institutions are formally supporting One Health approaches in country implementation and investments [17,18,19]. The World Health Organization (WHO), World Organisation for Animal Health (OIE), and Food and Agriculture Organization (FAO) formed the Tripartite agreement in 2010 to work closely together on AMR, rabies, and zoonotic influenzas, as well as zoonotic tuberculosis and Middle East Respiratory Syndrome-Coronavirus (MERS-CoV). The Tripartite also provides standard tools and guidance for taking a multisectoral, One Health approach to zoonoses and other health threats at the human-animal-environment interface, including The Tripartite Zoonoses Guide which has a dedicated chapter on workforce development [19]. The United Nations (UN) Convention on Biological Diversity’s focus on biodiversity extends to benefits for human and animal health. A One Health Operational Framework published by the World Bank in 2018 provides an overview and compendium of work to date in the context of international development, with examples of key initiatives and entities, implementation guidance, and an annex of resources [16]. Capacity assessment tools such as WHO’s Joint External Evaluation and OIE’s Performance of Veterinary Services are a basis for human and animal health system strengthening, potentially involving One Health strategies [1]. Several agencies in the U.S. have One Health webpages with a variety of resources, including the Centers for Disease Control and Prevention (CDC) which established a One Health Office in 2009 [20,21,22].

The number of Massive Open Online Courses (MOOCs) and other online educational resources on One Health is growing, providing an unprecedented opportunity for students and professors to more equitably access high quality content. For example, the MOOC “Global Health at the Human-Animal-Ecosystem Interface” (freely available on Coursera) presents One Health as a central approach in global health, with perspectives and applications from a diversity of experts and organizations [23]. Several public and global health conferences, including the annual meeting of the Consortium of Universities for Global Health, now include One Health content, though sometimes under different headings (e.g. environmental health, planetary health, and veterinary public health). The CDC hosts a free monthly webinar called the Zoonoses and One Health Updates (ZOHU) Call with free continuing education for physicians, veterinarians, and other professions [24]. In addition to specialized journals (e.g., *EcoHealth, One Health, The Lancet Planetary Health, One Health Outlook)*, global health and medical journals have published papers involving One Health approaches related to disease dynamics and operational risk reduction strategies [25,26,27,28].

These resources are commonly utilized by many actors implementing One Health approaches in their work, but awareness of them is limited in medical and global health communities. Although not easy given the structure of many medical school programs, inclusion of this information in curriculum, clinical lectures and continuing education opportunities is needed to increase familiarity and interest among medical professionals. Innovative pedagogical models and assessment methods could assist; for example, a Global Flipped Classroom on One Health interacted with a MOOC by viewing it and then developing interdisciplinary team-based One Health projects that received expert feedback, providing a blended-learning and research-oriented educative event [29,30].

Physicians must be prepared to routinely incorporate the One Health approach in work-up of infectious disease patients with animal and/or environmental exposures [31,33,32]. Posting key links on school websites, in course syllabi or lectures, or by enlisting school librarians can direct faculty and students to useful resources. Existing resources in the medical community, such as Medscape, could also devote space to One Health considerations (e.g., in disease summaries or continuing education) to expand awareness of ecological and epidemiological links relevant to disease transmission.

### 2) Creating Pathways for Information Flow in Clinical Medicine and Global Health Practice

Relevant entry points for medical and global health practice should build on One Health education. The dissemination of information beyond ones’ immediate sphere of practice is at the foundation of One Health. Identification of zoonoses by astute clinicians is a key pathway for early detection and effective rapid response to disease. These front-line observations of clinicians, veterinarians, and other health practitioners are invaluable but rely on dedicated awareness, time, effort, and optimal communication channels to guide appropriate response. Recognizing the power of the clinician for disease detection at the frontline of emergence, the USAID-funded PREDICT Project empowered health care providers in more than 30 low- and middle-income countries by connecting them with animal and environmental health professionals. Together they collect data on infectious disease transmission risks and unexpected and novel viruses that could be pathogenic in their communities [33].

While information flow can quickly become unwieldy, focused information channels can provide high value. ProMED-mail, the International Society for Infectious Diseases Program for Monitoring Emerging Diseases, aggregates media, first-hand and official reports of disease in humans, animals, and plants and provides interpretation by expert moderators. This program is complemented by a wider network through EpiCore, both of which provide real-time access to experts around the world for rapid outbreak and exposure determination. The freely available Web site “healthmap.org” and mobile app “Outbreaks Near Me” also deliver real-time intelligence on a range of emerging infectious diseases for a diverse audience including libraries, local health departments, governments, and international travelers.

Information from other disciplines is increasingly targeted to public health and medical settings; for example, climate and vegetation data from Geographical Information Systems (GIS) and satellite imagery monitoring are being channeled to public and animal health authorities, informing early warning and forecasting systems for vector-borne diseases such as Rift Valley fever and dengue. Use of these data can guide preventive vaccination strategies and preparation efforts to preempt disease threats and monitor control programs. More broadly, information sharing may increase statistical power and improve resource efficiency through enhanced understanding and targeting of risks and coordination of efforts [34,35]. Multisectoral, One Health coordination mechanisms in countries are facilitating new avenues for information sharing across sectors [19]. Ensuring that information channels are designed, operationalized, and maintained with workflow needs and utility for potential users (including clinicians) in mind can drive value and participation.

### 3) Developing Innovative Partnerships for Health Sector Impacts and Benefits

Clinicians frequently consult with and refer to other health specialists and understand the benefits from these interdisciplinary exchanges. Building on this approach to involve other disciplines can bring targeted information, understanding, and action to enhance disease detection and preventive medicine, improve access to care, guide implementation of health programs, and assist with behavior change. Several academic institutions have established novel One Health programs to link students and faculty from different departments, from research to service delivery, through a variety of mechanisms. Examples include joint degrees, events coordinated by medical, veterinary, and public health schools, leadership experiences involving scenario-based case discussions, and research or practicum exchanges [29,30,36,37].

One Health partnerships offer practical applications and advantages for clinicians, with potential impacts and benefits in patient care, research, and academia. One Health collaborations may keep clinicians abreast of disease risks translating to differential diagnoses and source attribution for diseases linked to environmental or animal factors (e.g., leptospirosis associated with flooding events, respiratory illness from toxic algal blooms, reptile or amphibian pet ownership associated with *Salmonella*). Delivery of preventive care is another area where One Health collaboration is already practiced to some extent through vaccination for patients traveling to disease-endemic areas and advice on exposure avoidance. Animal-assisted and nature-based therapies and benefits of the human-animal bond could have increasing applications in healthcare and uptake of healthy behavior [38,39]. Veterinary reporting of active animal disease events can guide real-time public health measures; detection of canine rabies, for example, informs post-exposure prophylaxis in human contacts. Similarly, the first detection of West Nile virus in the U.S. was by Bronx Zoo staff in birds while human encephalitis cases were being investigated by public health authorities [40,41]. Linking the two events could have increased ability to rapidly counter the threat of a virus new to North America. One Health-informed assessment of health risks for a patient population can spark heightened awareness by providers and health facilities, allowing for activation of preparedness protocols such as infection prevention and control, stockpiling, networks of disease specialists, and risk communication.

One Health partnerships in research allow the health sector to incorporate a broader set of variables for more complete risk assessment and management. They can assist in anticipating effects of changes outside of the health system (e.g., climate change) to mitigate poor outcomes. Similarly, they can improve understanding of disease transmission cycles, characterize types and relevance of exposure risks, and identify indicators for human outcomes. Coordination among multiple sectors can inform design and resource sharing for cost-effective risk management strategies, including animal health interventions (e.g. livestock vaccination) that deliver wider societal benefits like avoided cost of human disease [42]. Evaluation frameworks such as the Checklist for One Health Epidemiological Reporting of Evidence (COHERE) provide guidance on quality of reporting for observational studies and data integration from human, animal and environmental health sectors [43].

In resource-limited settings where critical infrastructure and transport systems are lacking, personnel, programs and resources in animal and environmental sectors can potentially be leveraged for health research, implementation activities, and surge capacity during emergencies. Other sectors may support trust building with relevant communities and stakeholders. Recent Ebola outbreaks demonstrate the importance of multi-sectoral expertise in risk-based behavior change campaigns; social sciences such as anthropology have guided appropriate community-backed solutions for dignified but safe burial and effective communication around wild meat consumption [44,45].

In academia, One Health partnerships can complement patient care skills to reinforce competencies for addressing population-level social, behavioral, economic, legal, and environmental determinants of health. These skill sets may enable practitioners to serve in a variety of clinical and non-clinical settings. For example, the Knights Landing One Health monthly clinic in California provides animal and human wellness services in an economically-disadvantaged community with low access to veterinary services and underserved healthcare needs [36]. The clinic was developed through collaboration between groups including the UC Davis Schools of Medical and Veterinary Medicine, community activists, and county agencies, and is primarily run by students – illustrating students’ desire to move in this direction. The clinic’s holistic approach has delivered vaccines and services (many for the first time for peoples’ animals), led to a joint diagnosis of scabies, and provides a more complete community health picture that is informing targeted health education [36].

Universities have ready access to scientific and other resources that support efficiencies in multi-disciplinary training and research initiatives. Even independent medical centers without links to other academic departments or networks can serve as models for One Health approaches, especially at community level. Partnerships can be developed on specific topic areas; for example, the “Many Hosts of Mycobacteria” conferences supported by the National Institute of Allergy and Infectious Diseases brings together researchers, clinicians, and veterinarians for focused interaction and comparative mycobacterial disease discussions. Diverse funding streams are available from a range of entities on topics that benefit from and invite broadened participation, such as federally-supported translational diagnostic development for zoonotic pathogens to foundations supporting community resilience against environmental health threats. Solutions may come from non-traditional actors; for example, the Jane Goodall Institute’s partnership with Microsoft to monitor mosquito diversity and abundance helps track vector-borne disease risks for great apes as well as humans living in proximity to their habitat.

Given the importance of the One Health approach to problem solving in educational programs, an expert group spanning plant sciences, human and veterinary medicine, and disease ecology developed core competencies that support collaboration among disciplines in development of curricula and programs [46]. Their review of past efforts to develop core competency domains and a survey of current educational offerings found no specific competency recommendations to guide curricular development, resulting in uneven coverage. The effort provided a blueprint for teams interested in incorporating One Health into expanded, transdisciplinary curricular offerings, suggesting three competency domains with 20 core competencies: Health Knowledge; Global & Local Issues in Humans, Animals, Plants, and the Environment; and Professional Characteristics. Recommendations for programs teaching the One Health approach emphasize a commitment to competency-based education that engages learners in practical training and communication for better coordination and collaborative problem solving. This recent work signals existing momentum and potential entry points, but dedicated efforts are needed to realize One Health coverage in curricula and practitioner competencies.

### 4) Informing and Empowering Health: The Critical Need to Inform the Public

Clinical or global health specialists can inform the public at all levels about a variety of global health challenges and evidence-based solutions. Understanding their professional roles in relation to One Health is critical to addressing social and environmental determinants of health and the Sustainable Development Goals (SDGs), from food security and poverty reduction to pandemic prevention and tackling non-communicable disease burden. Its multidisciplinary nature can unite sectors to address urgent environmental threats the planet is facing, including halting the sixth global extinction crisis we are undergoing, mitigating the impact of climate change, and protecting a range of ecosystem services including the neglected area of freshwater security. The approaches employed by the International Physicians for the Prevention of Nuclear War (IPPNW) provide an example of leadership in informing the public of risk. The IPPNW offers medical and scientific information on nuclear war, mobilizing a network of thousands of health workers and concerned citizens on the premise of physician obligation “to prevent what they cannot treat.” A similar approach could convey the importance of a One Health approach for urgent public health concerns under the obligation of “duty of care” to anticipate risks for patients or population served and take care to prevent them coming to harm.

Whether with individual patients or at local, state, federal, or international levels, healthcare workers have knowledge and credibility to speak about issues affecting optimal health status. With a One Health perspective they can articulate drivers, causes and effects threatening health and wellbeing, including extreme heat, flooding events, land desertification, deforestation, illegal wildlife trade, inadequate biosecurity, food and water insecurity, and poor antimicrobial stewardship. For example, surges in respiratory irritation that physicians see during red tide events can inform environmental monitoring strategies and regulations to reduce chemical runoff and protect critical water sources [47,48]. Threats and solutions can be framed through economic and security lenses often used for decisions affecting the public purse, aided by clear and easily understood messaging on actions and rationale.

## Conclusion

A basic appreciation of multisectoral and transdisciplinary approaches to individual and global health challenges is crucial for addressing complex health threats, including countering health consequences from societal and environmental challenges such as war, nutrition insecurity, pollution, loss of biodiversity and degraded ecosystem services, and climate change. One Health can be an effective platform to address both infectious and non-communicable diseases, the latter which are responsible for 70% of deaths worldwide [49].

The four objectives in this paper are intended as starting points to support medical and global health schools and centers interested in applying One Health approaches. They can leverage existing resources and infrastructure (e.g., virtual library services) to become standard practice. There are possible practical barriers and limitations to One Health engagement in clinical settings that need to be considered upfront for successful design, especially if expanded scope diverts attention from immediate care needs.

There is no one-size-fits-all approach to equip current and future global health professionals with all of the skills and competencies to meet the needs of patients and populations in the 21^st^ century; therefore, prioritization and using available resources efficiently is important. The One Health approach provides this opportunity as a powerful platform to create a healthier, sustainable world for all. We encourage medical and global health schools and practitioners to feel empowered to share their experiences, needs and recommendations to advance uptake and benefits of One Health approaches.

## Disclaimer

The authors alone are responsible for the views expressed in this article and they do not necessarily represent the views, decisions or policies of the institutions with which they are affiliated. Use of trade names and commercial sources is for identification only and does not imply endorsement by author institutions.
